# Genome-Wide Identification and Expression Analysis of the AS2/LOB Transcription Factor Family in *Asparagus officinalis*

**DOI:** 10.3390/genes16121411

**Published:** 2025-11-27

**Authors:** Xiao Ye, Yu Li, Sheng-Fu Zhong, Wei-Nian Huang, Jing Zeng, Qian Zuo, Shu Li, Pei Sun, Shan Tao, Ling Huang, Ming-Zhi Zhong, Wen-Ji Zhao, Yu-Xiang Shen, Yang Tao, Jie-Qiong Deng

**Affiliations:** 1 Industrial Crop Research Institute, Sichuan Academy of Agricultural Sciences, Chengdu 610300, China; troubadourelf666@gmail.com (X.Y.);; 2 School of Pharmacy, Chengdu Medical College, Chengdu 610500, China; 3 Horticultural Crops Germplasm Innovation and Utilization Key Laboratory of Sichuan Province, Sichuan Academy of Agricultural Sciences, Chengdu 610066, China; 4 Sichuan Academy of Grassland Sciences, Chengdu 611731, China; 5 School of Ecology and Environment, Tibet University, Lhasa 850000, China; 6 Agricultural College, Anshun University, Anshun 561000, China; 7 Institute for Advanced Study, Chengdu University, Chengdu 610106, China

**Keywords:** AS2/LOB, transcription factor family, drought response, *Asparagus officinalis*, expression profiling

## Abstract

Background: AS2/LOB transcription factors are central regulators of plant organ development and stress responses, yet their characteristics in the monocot crop *Asparagus officinalis* remain uncharacterized. Methods: In this study, we leveraged the *A. officinalis* genome to perform a genome-wide identification and comprehensive characterization of the AS2/LOB family. We identified 20 *AoAS* genes (*AoAS01*–*AoAS20*) and analyzed their physicochemical properties, chromosomal localization, conserved domains and motifs, phylogenetic relationships, gene structures, *cis*-regulatory elements, duplication history, syntenic relationships, protein–protein interaction networks and expression profiles. Results: Phylogenetic analysis divided the *Ao*AS proteins into two major clades (Class I and Class II), while chromosomal mapping revealed their uneven distribution across eight chromosomes. Analysis of publicly available RNA-seq data showed that 14 *AoAS* genes exhibit dynamic expression across four developmental stages of the stem (10, 25, 40 and 60 cm), with *AoAS11* and *AoAS14* consistently displaying high transcript levels. Under drought stress, 12 *AoAS* genes showed significant transcriptional changes, with *AoAS04* and *AoAS14* exhibiting the most pronounced expression responses. Conclusions: Together, these results provide a genome-wide portrait of the AS2/LOB family in asparagus, reveal their potential roles in development and drought response, nominate candidate genes for breeding stress-tolerant cultivars, and offer a useful benchmark for molecular breeding in economically important species including peony (*Paeonia lactiflora*).

## 1. Introduction

To build their complex structures, plants rely on master-switch genes that control development. A key group of these genes acts like architectural surveyors, defining the precise boundaries where organs like leaves and flowers will form. This critical group is known as the ASYMMETRIC LEAVES2/LATERAL ORGAN BOUNDARIES (AS2/LOB) gene family. The AS2/LOB family was first identified by enhancer trapping in *Arabidopsis thaliana* and represents a group of plant-specific transcription factors [1]. AS2/LOB proteins share a conserved ~100–amino-acid domain composed of three hallmark features: (1) a C-motif (CX_2_CX_6_CX_3_C) containing four cysteines essential for DNA binding; (2) a glycine–alanine–serine (GAS) block that contributes to maintaining structural and functional integrity; and (3) a leucine-zipper-like coiled-coil motif with an LX_6_LX_3_LX_6_L spacing that facilitates dimerization [2,3,4,5,6]. Phylogenetic analysis of these conserved domains divides the family into two major classes—Class I (further subdivided into Ia and Ib) and Class II. Class I members possess AS2/LOB domains closely resembling the canonical LOB proteins, whereas Class II members retain the conserved N-terminal region but diverge more extensively outside the core domain [7]. Genome-wide surveys have since cataloged AS2/LOB family members across diverse plant species, including *A. thaliana* (42 members), barley (*Hordeum vulgare*, 24), potato (*Solanum tuberosum*, 43), moso bamboo (*Phyllostachys edulis*, 55), turnip rape (*Brassica rapa*, 62), physic nut (*Jatropha curcas*, 28), and oilseed rape (*Brassica napus*, 126) [8,9,10,11,12,13,14]. Despite this diversity of identified family members and emerging functional insights, the identity, evolution, and stress-related roles of AS2/LOB genes in asparagus (*Asparagus officinalis* L.) remain uncharacterized.

Functional divergence within the AS2/LOB family is largely driven by variation in their C-terminal regions, as revealed in multiple species. In *A. thaliana*, *LBD37/38/39* act as conserved repressors of anthocyanin biosynthesis and nitrogen signaling [15]. In moso bamboo, overexpression of *PheLBD29* in *A. thaliana* reduces leaf size and induces abaxial curling while enhancing drought tolerance via increased soluble sugars and decreased malondialdehyde [16]. In lily, *LaLBD37* promotes bulb organ primordia initiation through carbohydrate–hormone crosstalk [17]. In chrysanthemum, *CmLBD2* regulates sporopollenin biosynthesis by binding the *CmACOS5* promoter; its suppression disrupts tapetum degradation and pollen development [18]. In tea (*Camellia sinensis*), *CsLBD37* causes dwarfism, early flowering, and reduced nitrate accumulation when expressed in *A. thaliana* [19]. Stress-related functional divergence is also evident: *MdLBD3* from apple enhances salinity and drought tolerance and accelerates flowering in *A. thaliana* [20], whereas *VvLBD39* from grape acts as a negative regulator, increasing sensitivity to PEG6000/NaCl and reducing drought/salinity tolerance and ABA responsiveness in heterologous systems [21]. These examples underscore both conserved and context-dependent roles of AS2/LOB members in development and stress adaptation.

Asparagus is a perennial herbaceous plant cultivated for over 2000 years as both a vegetable and a medicinal crop. It is rich in bioactive compounds—including saponins, flavonoids, and polysaccharides—and exhibits antioxidant, anticancer, anti-inflammatory, immunomodulatory, hypoglycemic, and hypolipidemic activities [22,23]. With accelerating global climate change and intensifying abiotic stresses such as drought, soil salinization, and high temperature, elucidating the molecular mechanisms of stress response and breeding stress-resilient asparagus has become increasingly urgent. Several gene families, including NAC, AUX/IAA, HSF and bZIP, have been implicated in asparagus abiotic stress responses [24,25,26]. However, despite extensive characterization of AS2/LOB genes in other species and their established links to stress adaptation, their roles in asparagus remain poorly documented.

In this study, we conducted a genome-wide identification and characterization of the AS2/LOB gene family in asparagus, including analyses of physicochemical properties, chromosomal localization, conserved domains and motifs, phylogenetic relationships, gene structures, *cis*-regulatory elements, duplication history, syntenic relationships, protein–protein interaction networks and expression profiles. We also leveraged publicly available RNA-seq data to profile *AoAS* gene expression in asparagus stems across four developmental stages and under drought stress. The resulting dataset provides a theoretical framework and resource for cloning and functional studies and offers candidate genes for breeding asparagus cultivars with improved quality and drought tolerance.

## 2. Materials and Methods

### 2.1. Identification and Analysis of the Physicochemical Properties of AoAS Members

The genome sequence, annotation file, protein sequences, and coding sequences (CDS) of asparagus used in this study were sourced from the National Center for Biotechnology Information (NCBI, https://www.ncbi.nlm.nih.gov/) under the accession number GCF_001876935.1 (accessed on 4 December 2024). The *A. thaliana* AS2/LOB protein sequences from Araport11 were downloaded from TAIR (https://www.arabidopsis.org/) (accessed on 1 March 2025) and the AS2/LOB protein domain Hidden Markov Model (HMM) PF03195 was obtained from the Pfam database (http://pfam.xfam.org/) (accessed on 1 March 2025). To screen candidate AS2/LOB sequences of asparagus, both HMMER 3.3 and the local BLAST+ 2.15.0 were utilized with the ‘10 × 10^−5^’ E-value parameter to search for sequences containing the AS2/LOB protein domain [27,28]. Following this, the identified protein sequences were uploaded to the Conserved Domain Database (CDD) (https://www.ncbi.nlm.nih.gov/Structure/bwrpsb/bwrpsb.cgi) (accessed on 1 March 2025) and SMART tool (http://smart.embl-heidelberg.de) (accessed on 1 March 2025) for sequence alignment and analysis. A total of 20 candidate AS2/LOB gene family members were identified in asparagus. Basic gene information, such as locus, strand, number of mRNA, number of exons, number of CDSs, and gene length, was compiled using TBtools-II [29]. The ExPASy Proteomics Server (https://www.expasy.org/) (accessed on 3 March 2025) was utilized to analyze the amino acid sequences and calculate their lengths, isoelectric points, and molecular weights [30]. Transmembrane helices were examined using TMHMM 2.0 (https://services.healthtech.dtu.dk/services/TMHMM-2.0/) (accessed on 3 March 2025) [31], while subcellular localization was predicted by DeepLoc-2.1 (https://services.healthtech.dtu.dk/services/DeepLoc-2.1/) (accessed on 3 March 2025) [32].

### 2.2. Structural Analysis and Chromosomal Localization of AoAS Genes

Information on 20 *AoAS* genes was extracted from the asparagus genome annotation file using TBtools-II, and gene structure diagrams were generated via the GSDS 2.0 online platform (https://gsds.gao-lab.org/) (accessed on 10 March 2025) [33]. The chromosomal distribution of the 20 *AoAS* genes across 10 chromosomes was visualized using the MG2C V2.1 online platform (http://mg2c.iask.in/mg2c_v2.1/index_cn.html) (accessed on 13 March 2025) [34].

### 2.3. Phylogenetic Analysis and Conserved Motif Characterization of AoAS Proteins

The amino acid sequences of 20 asparagus and 42 *A. thaliana* AS2/LOB family members were aligned using the ClustalW method implemented in MEGA-X software (Version 10.2.2) [35]. A phylogenetic tree was constructed using the Neighbor-Joining (NJ) method with a bootstrap value of 1000 and default parameters. Conserved motifs of *Ao*AS proteins were predicted using the MEME online platform (https://meme-suite.org/meme/) (accessed on 12 March 2025) with the following parameters: site distribution set to zero or one occurrence per sequence (zoops), number of motifs to 15, and motif width ranging from 6 to 50 amino acids [36]. Visualization was performed using TBtools-II, employing different colors to represent the 15 conserved motifs.

### 2.4. Cis-Acting Element Analysis of AoAS Gene Promoter Regions

Based on the CDS of AS2/LOB genes, TBtools-II was employed to align these sequences against the asparagus genome to retrieve the 2000 bp promoter sequences upstream of each gene’s start codon (ATG). Subsequently, the PlantCARE online tool (http://bioinformatics.psb.ugent.be/webtools/plantcare/html/) (accessed on 18 March 2025) was utilized to predict *cis*-acting elements within the *AoAS* gene promoter regions [37]. The results were visualized using TBtools-II.

### 2.5. Analysis of Duplication Events and Synteny of AoAS Genes

The duplication events of *AoAS* genes were predicted using the “One Step MCScanX—Super Fast” plugin in TBtools-II (E-value = 1 × 10^−3^, Number of Blast Hits = 10). The duplication type of each *AoAS* gene was subsequently classified. Among the 20 *AoAS* genes, three types of duplication were identified: dispersed, whole-genome duplication (WGD) or segmental, and tandem. The Ka/Ks ratios of tandemly duplicated gene pairs were calculated using the “Simple Ka/Ks Calculator” in TBtools-II based on the Nei-Gojobori method. The duplication patterns of *AoAS* genes were visualized with TBtools-II.

The genome sequences and annotation files of *A. thaliana*, *Dioscorea zingiberensis*, and *Solanum lycopersicum* were obtained from NCBI, while *Panax ginseng* data were downloaded from the National Genomics Data Center (https://ngdc.cncb.ac.cn/). The accession numbers were GCA_000001735.2 (accessed on 21 November 2024) for *A. thaliana*, GCA_014060945.1 (accessed on 14 November 2024) for *D. zingiberensis*, GCF_036512215.1 (accessed on 23 November 2024) for *S. lycopersicum* and GWHBEIL00000000.1 (accessed on 14 November 2024) for *P. ginseng*. Collinearity analysis between AS2/LOB genes in asparagus and these species was predicted using the “One Step MCScanX—Super Fast” plugin in TBtools-II (E-value = 1 × 10^−3^, Number of Blast Hits = 10). Results were visualized through TBtools-II’s graphic interface.

### 2.6. Analysis of AoAS Protein Interaction Network

A protein–protein interaction analysis was conducted for 20 *Ao*AS proteins using the STRING database (version 12.0; https://cn.string-db.org/) (accessed on 24 March 2025). *A. thaliana* was employed as the reference organism, with the required confidence score set to medium (0.400) and the false discovery rate (FDR) stringency set to medium (5 percent). These parameters were chosen to balance the inclusion of relevant interactions while minimizing potential false positives.

### 2.7. Expression Pattern Analysis of AoAS Genes

We retrieved RNA-seq data for asparagus stems at various developmental stages and under drought stress from public databases to analyze the expression profiles of *AoAS* genes. The transcriptomic data of asparagus stems at different developmental stages was obtained from the NCBI Gene Expression Omnibus (GEO) database under accession number GSE252560 (accessed on 9 April 2025). The samples were collected at four distinct developmental stages, corresponding to stem heights of 10, 25, 40 and 60 cm [38]. Each treatment was conducted with three biological replicates to ensure statistical reliability. The resulting data underwent standard bioinformatic processing to generate fragments per kilobase of transcript per million fragments mapped (FPKM) values as a measure of gene expression. The means of each gene’s expression across the three biological replicates were normalized using the log2(FPKM + 1) transformation to facilitate visual representation.

The RNA-seq data of asparagus (Pacific Early and Jilv3) leaves under control and drought stress conditions were obtained from 12 SRA files (SRX17208360-SRX17208371) in Project PRJNA873275 (accessed on 16 April 2025) from NCBI. In the control group, plants were watered every two days to maintain a soil water content of 75–80%, while the drought treatment group received no watering. Three biological replicates were set for each treatment [39]. Fastq paired-end data containing forward and reverse reads were generated from the SRA files. The quality of the reads was initially assessed using FastQC [40] and MultiQC [41], and adapter sequences and low-quality reads were trimmed using Trimmomatic [42]. The reads were then aligned to the reference genome using HISAT2 [43] to generate BAM files, and FPKM values for each gene were calculated using StringTie [44]. The expression means of each gene across the three biological replicates were normalized using min-max normalization and log2(FPKM + 1) transformation to facilitate visualization.

## 3. Results

### 3.1. Identification and Chromosome Localization of AS2/LOB Gene Family Members in Asparagus

A total of 20 AS2/LOB gene family members were identified in asparagus and designated *AoAS01*–*AoAS20* based on their sequential chromosomal localization. The amino acid sequences and physicochemical properties of all 20 *Ao*AS proteins are summarized in Supplementary Tables S1 and S2, respectively, including locus, strand, transcript number, exon count, CDS count and gene length, as well as protein length, number of transmembrane helices, theoretical pI, molecular weight and predicted subcellular localization. *AoAS17* (632 bp) and *AoAS12* (8138 bp) were the shortest and longest genes, respectively. AoAS08 contained the fewest amino acids (139 aa) and showed the lowest molecular weight (15,625.34 Da), in contrast to AoAS06, which had the highest amino acid count (238 aa) and largest molecular weight (26,151.45 Da). Transcript variants differed in mRNA number (1–3; *AoAS11* has 3), exon count (1–3; *AoAS06* has 3) and CDS number (1–2). Among these proteins, 9 were acidic (pI < 7.0) and 11 were alkaline (pI > 7.0). All *Ao*AS members exhibited zero transmembrane helices, with subcellular localization predictions exclusively indicating nuclear localization. These features of *Ao*AS family members—nuclear localization, absence of transmembrane helices, and a characteristic bimodal pI distribution—are consistent with those typically observed for plant transcription factors.

**Figure 1:**
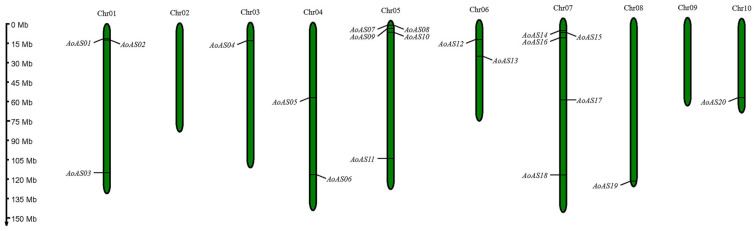
Distribution of 20 *AoAS* genes across 10 chromosomes in asparagus.

The chromosomal localization of the 20 *AoAS* genes was analyzed (Figure 1). The results showed that these genes are unevenly distributed across 8 out of the 10 chromosomes (Chr02 and Chr09 contain no *AoAS* genes). The highest gene density was observed on Chr05 and Chr07, each harboring five genes: *AoAS07–AoAS11* and *AoAS14–AoAS18*, respectively. Chr01 contains three genes (*AoAS01–AoAS03*), whereas Chr04 and Chr06 each contain two genes (*AoAS05*, *AoAS06* and *AoAS12*, *AoAS13*, respectively). Chr03, Chr08, and Chr10 each contain a single gene (*AoAS04*, *AoAS19* and *AoAS20*, respectively). Notably, approximately 70% of the genes were located at the chromosomal termini. Furthermore, *AoAS07* and *AoAS08* on Chr05 were identified as tandemly duplicated genes, with an intergenic distance of 1559 bp.

### 3.2. Analysis of the Amino Acid Sequence of the Conserved Domain and Motifs in AoAS Proteins

**Figure 2:**
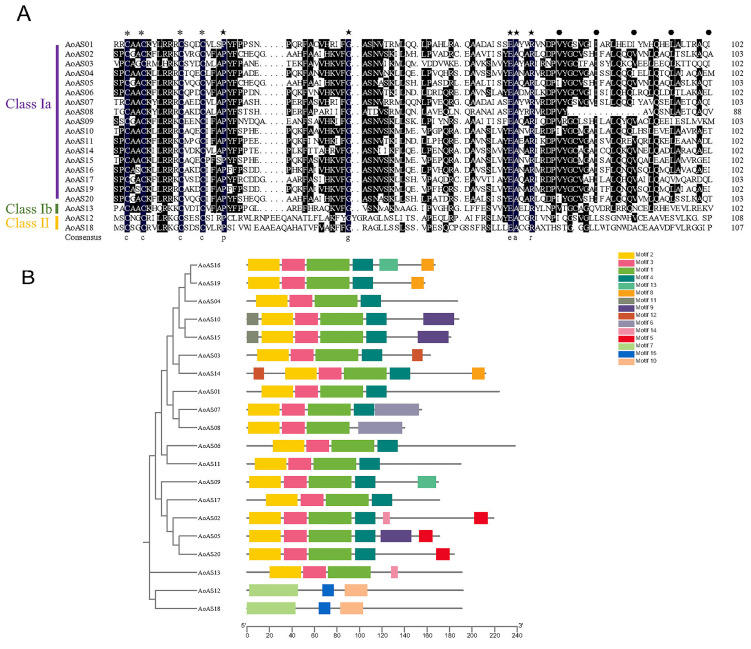
Sequence alignment and conserved motif architecture of *Ao*AS proteins. (**A**) Multiple sequence alignment of *Ao*AS proteins. The consensus sequence of the C-motif is indicated by asterisks (*), and hydrophobic residues in the LZ-like region are marked with black dots (●). Amino acid residues conserved in more than 10 members of each group are shown as white letters on a black background, whereas pentagrams (★) indicate residues that were strictly conserved across all *Ao*AS proteins. (**B**) Conserved motif architecture of *Ao*AS proteins. Motifs were predicted using MEME and visualized with TBtools-II; the 15 identified conserved motifs are represented by differently coloured boxes.

The conserved characteristics of the AS2/LOB domain in 20 *Ao*AS proteins were analyzed using DNAMAN 6.0 software. Based on the amino acid sequences of *A. thaliana* AS2/LOB [1], these proteins were classified into three subgroups: Class Ia (containing 17 proteins), Class Ib (containing 1 protein), and Class II (containing 2 proteins), as shown in Figure 2A. The CX_2_CX_6_CX_3_C sequence (where X represents non-conserved residues and subscript numbers indicate the number of non-conserved residues), hereafter referred to as the C-motif, was strictly conserved across all identified asparagus AS2/LOB protein domains. In addition to the C-motif, the leucine zipper-like (LZ-like) sequence was also highly conserved among these proteins. More than half of the proteins contained the following conserved sequences: CXACKXLRRXCX_3_C within the C-motif, FX_3_HKVFG..ASNVXKXL between the C-motif and the LZ-like sequence (“.” denotes a gap in the amino acid sequence), and YGCX_3_I and LQXQ within the LZ-like sequence. Additionally, five amino acid residues (P20, G43, E73, A74 and R77, where the numbers indicate the residue positions) were strictly conserved in the AS2/LOB domains of all 20 *Ao*AS proteins.

An analysis of conserved motifs was conducted on the amino acid sequences of 20 *Ao*AS proteins (Figure 2B), with each motif’s primary sequence detailed in Supplementary Table S3. Fifteen distinct motifs were identified, and their distribution patterns diverged markedly between Class I and Class II members. Class I proteins uniformly adopted a Motif 2–Motif 3–Motif 1 topology, whereas Class II proteins consistently exhibited a Motif 7–Motif 15–Motif 10 configuration. On the basis of sequence features, we infer that Motif 2 and Motif 7 represent the C-motifs of Class I and Class II, respectively, while Motif 1 and Motif 10 correspond to leucine-zipper-like regions in their respective classes. Motif 3 was conserved across all Class I proteins and Motif 4 was predominantly present in the Class Ia subgroup. Several motifs displayed branch specificity: Motif 13 occurred exclusively in *Ao*AS16 and *Ao*AS09; Motif 8 in *Ao*AS16, *Ao*AS19, and *Ao*AS14; Motif 9 in *Ao*AS10, *Ao*AS15, and *Ao*AS05; Motif 14 in *Ao*AS02 and *Ao*AS13; and Motif 5 in *Ao*AS02, *Ao*AS05, and *Ao*AS20. Moreover, proteins within individual phylogenetic clades shared unique motif signatures: Motif 11 appeared only in *Ao*AS10 and *Ao*AS15; Motif 6 only in *Ao*AS07 and *Ao*AS08; and Motif 15 only in *Ao*AS12 and *Ao*AS18.

### 3.3. Phylogenetic Analysis of AS2/LOB Proteins

**Figure 3:**
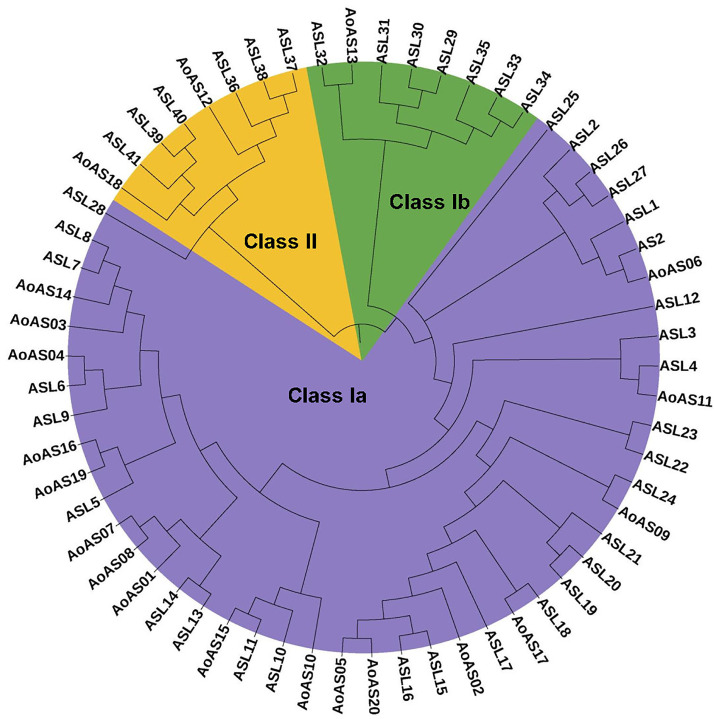
Phylogenetic analysis of *Ao*AS proteins. The neighbor-joining phylogenetic tree illustrates the evolutionary relationships among 42 AS2/LOB proteins from *A. thaliana* and 20 from asparagus. These proteins are clustered into three groups: Class Ia (orange), Class Ib (green), and Class II (purple).

Based on the phylogenetic trees of AS2/LOB proteins in *A. thaliana* and physic nut [1,8], we constructed a phylogenetic tree using 42 *A. thaliana* and 20 asparagus AS2/LOB proteins (Figure 3). These 62 proteins were grouped into two major clades, with Class I further divided into two subgroups. The Class Ia subgroup consisted of 29 AS2/LOB proteins in *A. thaliana* and 17 *Ao*AS proteins (*Ao*AS01–*Ao*AS11, *Ao*AS14–*Ao*AS17, *Ao*AS19 and *Ao*AS20), while the Class Ib subgroup included 7 AS2/LOB proteins in *A. thaliana* and 1 *Ao*AS protein (*Ao*AS13). The Class II subgroup comprised 6 AS2/LOB proteins in *A. thaliana* and 2 *Ao*AS proteins (*Ao*AS12 and *Ao*AS18). Furthermore, a total of seven *Ao*AS proteins formed sister pairs with AS2/LOB proteins in *A. thaliana*. These sister pairs included ASL32 and *Ao*AS13, ASL2 and *Ao*AS06, ASL4 and *Ao*AS11, ASL24 and *Ao*AS09, ASL18 and *Ao*AS17, ASL11 and *Ao*AS15, and ASL6 and *Ao*AS04.

### 3.4. Analysis of the Structure of AoAS Genes

**Figure 4:**
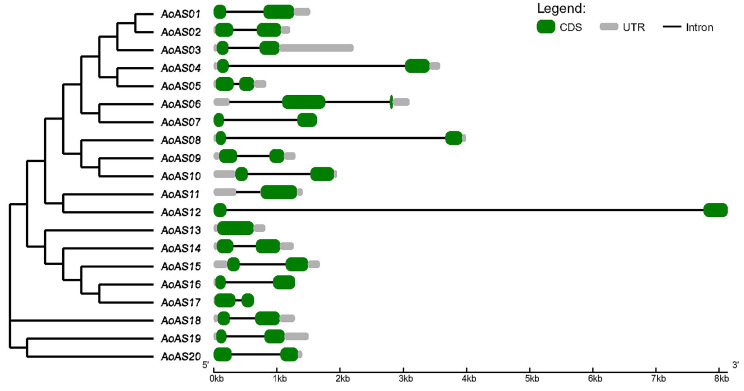
Gene structure of *AoAS* genes. CDS is represented by green rectangle, intron by black line, and UTR by gray rectangle.

The intron-exon structure of the 20 *AoAS* genes is illustrated in Figure 4. Most members encode two CDSs, while *AoAS11* and *AoAS13* have only one. The majority of the genes have a single intron, except for *AoAS06*, which contains two, and *AoAS13*, which has none. Most introns are relatively short, whereas those of *AoAS12* and *AoAS08* exceed 3 kb. The 5′ and 3′ untranslated regions (UTRs) are generally short across most members, except for *AoAS03*, which has an extended 3′ UTR region.

### 3.5. Analysis of the Promoter Elements of AoAS Genes

**Figure 5:**
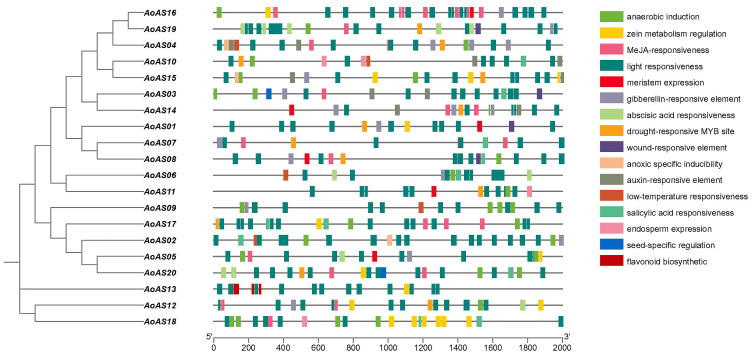
Analysis of *cis*-acting elements of *AoAS* genes, where different colored squares represent various functions.

The analysis of *cis*-acting elements within the 2000 bp promoter sequences upstream of the start codon of *AoAS* genes is presented in Supplementary Table S4 and Figure 5. These elements are categorized into three modules based on their functions: environmental signal responsiveness, phytohormone responsiveness, and tissue and development-specific regulation. Each module encompasses several subcategories. Within the environmental signal responsiveness module, elements associated with light responsiveness—such as ATCT-motif, Box 4, G-Box, GATA-motif, LAMP-element, MRE, and TCT-motif—are present in every *AoAS* gene and occur in substantial numbers. Additionally, elements related to anaerobic induction (ARE) are commonly found across most *AoAS* genes, suggesting that this gene family may be regulated by light signals and could have specific expression patterns under hypoxic conditions. This observation aligns with *cis*-element analyses conducted in *B. rapa* [9]. Furthermore, drought-responsive MYB site elements (MBS) also appear in more than half of the genes. In the phytohormone responsiveness module, gibberellin-responsive elements (TATC-box, P-box, GARE-motif) and MeJA-related elements (TGACG-motif) are present in over half of the genes, indicating that *AoAS* genes may play roles in plant defense mechanisms or responses to injury. The tissue and development-specific regulation module contains fewer distributed genes compared to the other two modules, with most subcategories scattered across different genes. Notably, flavonoid biosynthetic-related elements (MBSI) are exclusively found in *AoAS13*, and zein metabolism regulation-related elements (O2-site) are abundantly present in *AoAS18*, with up to eight occurrences.

### 3.6. Duplication Event and Synteny Analysis of AoAS Genes

**Figure 6:**
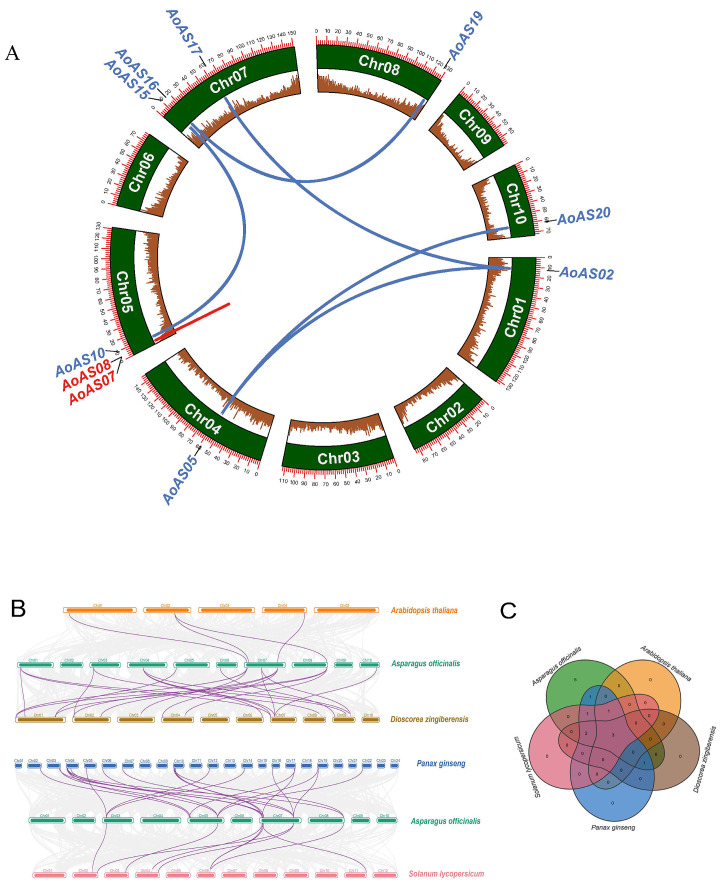
(**A**) Schematic representation of duplication patterns of *AoAS* genes. Red lines indicate tandem duplications between *AoAS* gene pairs, while blue lines represent WGD or segmental duplications. The brown bars denote gene density across the genome. (**B**) Syntenic relationships of AS2/LOB genes between *A. officinalis* and *A. thaliana*, *D. zingiberensis*, *P. ginseng* and *S. lycopersicum*. Gray lines connecting chromosomes of different species indicate all collinear blocks, while magenta lines specifically highlight the homologous relationships among AS2/LOB genes. (**C**) Venn diagram illustrating the shared and species-specific AS2/LOB genes among the five species.

Duplication events among the 20 *AoAS* genes were systematically analyzed, as presented in Supplementary Table S5 and Figure 6A. Among these, only one tandemly duplicated gene pair was identified: *AoAS07* and *AoAS08*, both located on Chr05. The non-synonymous to synonymous substitution ratio (Ka/Ks) for this gene pair was calculated to be 0.354513. Since this value is less than 1, the duplicated genes are likely under purifying (negative) selection, indicating that functional constraint was retained post-duplication [45]. Moreover, the relatively low Ks value (0.333101) implies that this tandem duplication event likely occurred in a relatively recent evolutionary period [46]. Five pairs of WGD or segmental duplicated genes were identified: *AoAS02–AoAS05*, *AoAS02–AoAS17*, *AoAS05–AoAS20*, *AoAS10–AoAS15* and *AoAS16–AoAS19*. These tandem and WGD or segmental duplication events were distributed across six chromosomes (Chr01, Chr04, Chr05, Chr07, Chr08 and Chr10). The remaining 10 genes (*AoAS01*, *AoAS03*, *AoAS04*, *AoAS06*, *AoAS09*, *AoAS11*, *AoAS12*, *AoAS13*, *AoAS14* and *AoAS18*) were classified as dispersed duplicates.

Further analysis of the syntenic relationships of AS2/LOB genes between asparagus and *A. thaliana*, *D. zingiberensis*, *P. ginseng* and *S. lycopersicum* was performed to identify homologous gene pairs (Figure 6B). A total of 20 *AoAS* genes exhibited collinearity with AS2/LOB genes from the four other species: 3 *A. thaliana* genes with 4 *AoAS* genes, 18 *D. zingiberensis* genes with 12 *AoAS* genes, 22 *P. ginseng* genes with 9 *AoAS* genes and 7 *S. lycopersicum* genes with 7 *AoAS* genes. These findings suggest that asparagus shares the highest degree of homology with *D. zingiberensis* and the lowest with *A. thaliana*. In addition, three conserved AS2/LOB genes were found to be present across all five species, corresponding to *AoAS14*, *AoAS18* and *AoAS19* in asparagus.

### 3.7. Analysis of the Interaction of AoAS Proteins

**Figure 7:**
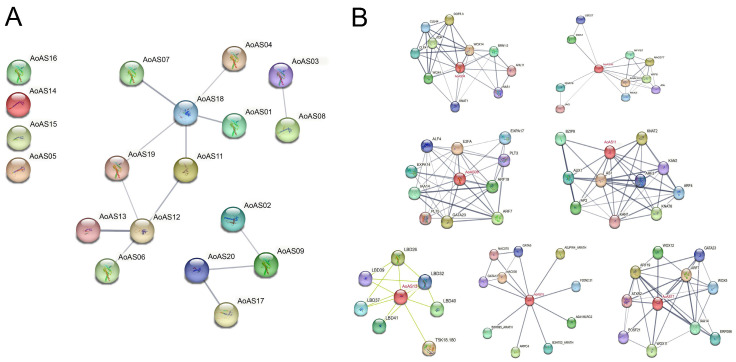
Protein–protein interaction (PPI) networks of *Ao*AS proteins predicted by STRING. (**A**) PPI network among all 20 *Ao*AS proteins in *A. officinalis*. (**B**) Predicted PPI networks centred on selected *Ao*AS proteins (*Ao*AS04, *Ao*AS06, *Ao*AS09, *Ao*AS11, *Ao*AS13, *Ao*AS15 and *Ao*AS17) showing their putative interacting partners. In both panels, edge thickness reflects interaction confidence, and the thickness of the edges represents the predicted interaction confidence; thicker lines indicate a higher probability of protein–protein interaction.

Using *A. thaliana* as a model species, the protein–protein interaction (PPI) network of *Ao*AS proteins was predicted via the STRING database, as shown in Figure 7 and Supplementary Table S6. Figure 7A illustrates that a total of 13 potential protein interaction pairs were identified. Among them, *Ao*AS18 appeared to be the central hub, interacting with five other proteins: *Ao*AS01, *Ao*AS07, *Ao*AS04, *Ao*AS19, and *Ao*AS11. *Ao*AS12 also showed extensive interactions with four proteins: *Ao*AS06, *Ao*AS11, *Ao*AS13, and *Ao*AS19. Notably, the interaction between *Ao*AS13 and *Ao*AS12 exhibited the highest confidence, with a combined score of 0.791. In contrast, *Ao*AS05, *Ao*AS14, *Ao*AS15, and *Ao*AS16 were not predicted to interact with any other proteins under the chosen cutoff (combined score ≥ 0.4).

Based on the phylogenetic analysis of AS2/LOB proteins (Figure 7B), the functions of seven asparagus proteins homologous to their *A. thaliana* counterparts were predicted. *Ao*AS04 interacts with WOX4, WOX14, TDR and CLE41/44, suggesting its role in regulating cell division and differentiation in vascular cambium [47,48,49]. *Ao*AS06 interacts with transcription factors KNAT6, RAX3, NAC077 and NFYB7, implicating its involvement in organ boundary establishment, meristem maintenance, and morphogenesis by coordinating transcriptional activities [50,51]. *Ao*AS09 interacts with auxin signaling components (ARF7/19, IAA14), cell wall modifiers (EXPA14/EXPA17) and meristem regulators (PLT3/PLT5), indicating its potential role in coordinating auxin-mediated vascular development [52,53,54]. *Ao*AS11 interacts with KAN1, KAN2, ARF3, AUX1, AP2 and BZIP8, suggesting its participation in shoot apical meristem maintenance, organ boundary formation, vascular differentiation, and auxin signal integration [55,56]; *Ao*AS13 establishes robust interactions with multiple LBD family proteins (such as LBD26, LBD39, and LBD32), potentially contributing to the development of lateral organ boundaries. *Ao*AS15 interacts with NAC075/NAC030 and GATA5/GATA12, potentially regulating stress responses, cytoskeleton organization and cell morphology [24,57]. *Ao*AS17 interacts with root development-associated proteins WOX11/12, ARF7/19 and IAA14, positioning it as a key node linking auxin signaling and the WOX regulatory network [58,59]. Collectively, these *Ao*AS proteins orchestrate critical physiological processes in asparagus, including vascular development, organ boundary patterning, auxin signaling, and stress response, through interaction networks with diverse regulatory partners.

### 3.8. Analysis of the Expression Pattern of AoAS Genes in the Stems of Asparagus at Different Growth Stages and Under Drought Stress

**Figure 8:**
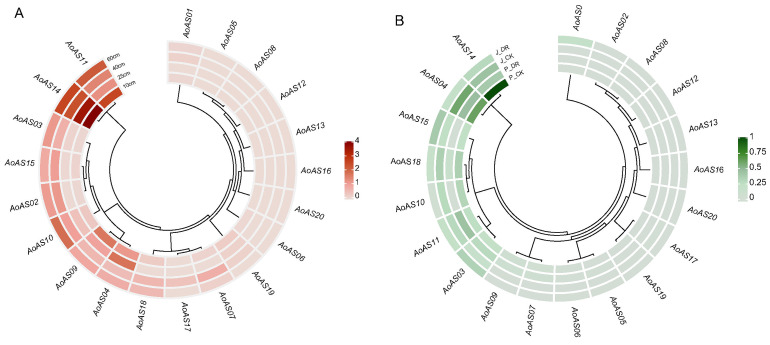
(**A**) Heatmap of the temporal expression profiles of *AoAS* genes in asparagus stems across different growth stages (stem heights: 10, 25, 40, and 60 cm), shown using a continuous red gradient (deeper red indicates higher expression; lighter red indicates lower expression). (**B**) Heatmap illustrating the expression patterns of *AoAS* genes under drought stress, using a continuous green gradient (deeper green indicates higher expression; lighter green indicates lower expression). J_DR and J_CK refer to drought-treated and control samples of the cultivar Jilv3, respectively; P_DR and P_CK refer to drought-treated and control samples of the cultivar Pacific Early, respectively.

To investigate the temporal expression characteristics and potential functions of *AoAS* genes, we analyzed transcriptome data derived from the stems of asparagus at different developmental stages (stem heights of 10, 25, 40 and 60 cm) [38]. The results revealed substantial variation in the expression of 14 *AoAS* family members across stem samples of varying heights (Figure 8A; Supplementary Table S7A). Some genes, such as *AoAS05*, *AoAS08*, *AoAS12*, *AoAS13*, *AoAS16* and *AoAS20*, were barely expressed at any stage, whereas others were active across multiple growth stages. *AoAS11* and *AoAS14* exhibited consistently high expression levels in all tissues, suggesting their involvement in broad developmental and regulatory processes. *AoAS14* displayed the highest overall expression, with a gradual decrease over time, indicating a potential role in early-stage stem development. Similarly, *AoAS19* showed progressively reduced expression, ultimately becoming undetectable at later stages. In contrast, *AoAS03* and *AoAS10* displayed a marked increase in expression over time, implying potential involvement in stem maturation or secondary metabolite accumulation. In addition, *AoAS06* was exclusively expressed at the 10 cm stage, while *AoAS17* was detected only at 40 cm.

To investigate the expression characteristics of *AoAS* genes under drought stress, we analyzed RNA-seq data focusing on 20 *AoAS* genes in the leaves of two asparagus cultivars (Jilv3 and Pacific Early) under control and drought stress conditions [39]. The results revealed that a total of 14 *AoAS* genes were expressed under drought stress, while six genes (*AoAS02*, *AoAS08*, *AoAS12*, *AoAS13*, *AoAS16* and *AoAS20*) were not detected in either cultivar under control and drought stress conditions (Figure 8B and Supplementary Table S7B). Conversely, seven genes (*AoAS03*, *AoAS04*, *AoAS10*, *AoAS11*, *AoAS14*, *AoAS15* and *AoAS18*) were expressed in both cultivars under control and drought stress conditions, with *AoAS04* and *AoAS14* exhibiting relatively higher expression levels. Notably, *AoAS03*, *AoAS05* and *AoAS06* were upregulated in both cultivars under drought stress, whereas *AoAS04*, *AoAS10*, *AoAS14* and *AoAS18* were downregulated. These results show that approximately 60% of *AoAS* genes respond to drought stress, with cultivar-specific expression differences, with some family members showing pronounced up- or down-regulation under drought conditions.

## 4. Discussion

In this study, we identified a total of 20 *AoAS* genes in the genome of asparagus (Figure 1 and Supplementary Table S2), which is substantially fewer than the numbers reported for the AS2/LOB family in several other plant species—for example, *A. thaliana* (42), *B. rapa* (62), *P. edulis* (55), *S. tuberosum* (43), and *B. napus* (126). Such variation in family size likely reflects differences in evolutionary history and the extent of genome duplication events across lineages. Gene family expansion is typically driven by duplication mechanisms including WGD, segmental duplication, and tandem duplication, whose frequency and retention vary among taxa [14,45]. Indeed, numerous studies have attributed the expansion of AS2/LOB families in grasses, rice, maize, and *Arabidopsis* to such duplication events [60]. In contrast, although asparagus experienced ancient WGD events (Asparagales-α and Asparagales-β) early in its evolution, no recent polyploidization has been documented [61,62]. The absence of recent genome duplications may have constrained expansion of the AS2/LOB family in asparagus, resulting in its relatively small complement of *AoAS* genes.

Consistent with observations in other plants, nearly all *AoAS* genes exhibit a conserved structural organization of two coding sequence regions separated by a single short intron, with exceptions such as *AoAS06* (which contains two introns) and *AoAS12*/*AoAS08* (which harbor unusually long introns) (Figure 4) [4,8,9,12,13,63]. The streamlined exon–intron architecture of AS2/LOB genes is thought to enable rapid transcriptional responses to developmental and environmental cues by facilitating efficient transcription and splicing [64,65]. The particularly long introns of *AoAS12* and *AoAS08*, coupled with their clustering in promoter element analyses, suggest that they may harbor additional *cis-*regulatory motifs influencing expression dynamics [66]. In contrast, the presence of two introns in *AoAS06* raises the possibility of alternative splicing, potentially generating isoforms with tissue-specific or stress-responsive roles and adding regulatory plasticity [67].

Promoter analysis further revealed that *AoAS* genes possess a multilayered *cis-*regulatory architecture integrating environmental (light, oxygen, drought), hormonal (gibberellin, methyl jasmonate), and developmental signals (Figure 5; Supplementary Table S4), consistent with the complexity required for fine-tuning nitrogen metabolism and environmental adaptation [68]. The unique occurrence of the MBSI element in *AoAS13* and the enrichment of O2-site elements in *AoAS18* point toward potential subfunctionalization within the family, whereby paralogs acquire distinct regulatory features to specialize in particular developmental contexts or organs [69]. Dissecting these individual *cis-*element contributions will facilitate rational design of synthetic promoters for precise spatial–temporal gene expression, as successfully implemented in crops such as rice and maize [25,70,71,72].

Duplication pattern analysis indicated that 50% of *AoAS* genes are derived from dispersed duplication, whereas only one tandemly duplicated pair (*AoAS07*/*AoAS08*) was identified (Figure 6A; Supplementary Table S5). The asparagus genome is rich in TEs, comprising approximately 53% of its content, with LTR retrotransposons predominant [62,73]. High-density TE landscapes can suppress non-allelic homologous recombination (NAHR), thereby limiting the formation of tandem duplicates and favoring dispersed retention [74,75,76,77]. The low Ka/Ks ratio (0.354513) of the *AoAS07*/*AoAS08* pair suggests strong purifying selection preserving their function post-duplication [78,79]. More broadly, most angiosperm AS2/LOB genes similarly originate from WGDs and dispersed duplications, with tandem clusters being rare [80]. Examples include limited tandem duplication in *A. thaliana* (three pairs among 42 members), *B. rapa* (five among 62), and *P. edulis* (two among 55), whereas *B. napus*—despite harboring 126 AS2/LOB genes—lacks detectable tandem duplicates [9,13,14,81]. This prevalent dispersed retention may reflect evolutionary constraints to minimize functional redundancy or expression interference, as spatial separation (different chromosomes) enables independent regulation and reduces genomic instability associated with tandem arrays [82,83].

Comparative synteny analysis further clarified evolutionary relationships: asparagus shares the most collinear AS2/LOB gene pairs with the monocot *D. zingiberensis* and the fewest with the eudicot *A. thaliana* (Figure 6B), consistent with broader angiosperm phylogenetic relationships [84,85]. Notably, both asparagus and *D. zingiberensis* predominantly accumulate steroidal saponins, whereas *P. ginseng* is enriched in triterpenoid saponins and *S. lycopersicum* and *A. thaliana* lack steroidal saponins [86,87,88,89,90]. The congruence between metabolic profiles and AS2/LOB collinearity suggests potential involvement of conserved family members in secondary metabolite regulation. Three *AoAS* genes (*AoAS14*, *AoAS18* and *AoAS19*) are conserved across all five species, implying preservation of fundamental developmental roles. Phylogenetic placement of these genes alongside their respective orthologs—*AoAS14* with *ASL7*/*ASL8*, *AoAS18* with *ASL39*/*ASL41*, and *AoAS19* with *ASL5*—supports functional conservation, predicting roles in vascular cambium proliferation and secondary growth (*AoAS14*), nitrate responsiveness and anthocyanin regulation (*AoAS18*), and lateral organ development (*AoAS19*) [91].

Protein–protein interaction predictions revealed that *Ao*AS proteins assemble into a multilayered, feedback-regulated network coordinating key physiological processes, including vascular development, organ boundary specification, integration of auxin and other hormone signaling, and stress adaptation (Figure 7). This integrative regulatory framework underscores the centrality of the AS2/LOB family in transducing endogenous and environmental cues to shape growth and differentiation. Future experimental validation—such as yeast two-hybrid assays and targeted gene editing—will be instrumental in dissecting specific interaction functions and leveraging these insights for precision improvement of asparagus traits.

Spatiotemporal expression profiling demonstrated that *AoAS* genes exhibit pronounced developmental stage-specific dynamics (Figure 8A), in line with observations in other species [4,8,11,12,92,93]. *AoAS11* and *AoAS14* are consistently highly expressed across stem samples, suggesting foundational roles in cambial maintenance and xylem differentiation. *AoAS14′*s high early expression followed by decline implies a pivotal function in early stem development that is modulated as growth proceeds. By contrast, the upregulation of *AoAS03* and *AoAS10* with increasing stem length suggests involvement in later developmental processes such as secondary growth or cell wall and metabolite biosynthesis. Under drought stress, roughly 60% of *AoAS* genes respond with differential expression (Figure 8B), with some (*AoAS03*, *AoAS05*, *AoAS06*) induced—potentially contributing to antioxidant defense, osmotic adjustment, or cell wall remodeling—and others (*AoAS04*, *AoAS10*, *AoAS14*, *AoAS18*) repressed, reflecting a trade-off between growth and stress resilience. Similar downregulation of developmental AS2/LOB genes under stress has been reported in other systems [8,9].

In summary, the *AoAS* gene family is tightly integrated into the regulatory networks governing organ development and environmental adaptation in asparagus. Its conserved structural features, clarified evolutionary trajectories, and diversified expression responses make it a robust set of candidates for future functional validation and guiding molecular breeding aimed at improving asparagus growth and stress tolerance. Additionally, for high-value, ultra-large-genome crops such as *P. lactiflora* and *Paris polyphylla*, whose genomic resources remain nascent, the asparagus-based AS2/LOB gene-family paradigm provides a tractable route to systematically identify and characterize key families [94,95]. Analyses of membership, gene structure, *cis-*regulatory elements, duplication/expansion and expression can elucidate the genetic architecture of key traits and furnish candidate targets and a theoretical basis for targeted molecular breeding.

## 5. Conclusions

We performed a comprehensive genome-wide identification and characterization of the AS2/LOB transcription factor family in asparagus and identified 20 *AoAS* genes that cluster into Class I and Class II subfamilies. These genes were systematically evaluated across multiple dimensions, including physicochemical properties, chromosomal localization, conserved domains and motifs, phylogenetic relationships, gene structures, *cis-*regulatory elements, duplication history, syntenic relationships, protein–protein interaction networks and expression profiles. Although the *AoAS* genes share conserved structural features, their expression profiles are highly variable, implying functional diversification in organ development, environmental adaptation and tissue-specific regulation.

By providing the first genome-wide catalogue and expression atlas of AS2/LOB genes in *A. officinalis*, this work fills an important gap in the functional genomics of this economically important perennial vegetable. The integrated structural and expression evidence highlights specific *Ao*AS members as promising regulators of spear/stem development and drought response, thereby offering testable candidates for future functional studies. In turn, these findings deliver a foundational resource and concrete gene targets for molecular breeding of stress-resilient asparagus and may also inform the improvement of other high-value crops such as peony through comparative and translational genomics.

## Data Availability

The genome sequence, annotation file, protein sequences, and coding sequences (CDS) of asparagus used in this study were sourced from NCBI under the accession number GCF_001876935 (NCBI, https://www.ncbi.nlm.nih.gov/, accessed on 4 December 2024). The transcriptomic data of asparagus stems at different developmental stages was obtained from the NCBI GEO database under accession number GSE252560 (accessed on 9 April 2025). The RNA-seq data of asparagus (Pacific Early and Jilv3) leaves under control and drought stress conditions were obtained from 12 SRA files (SRX17208360-SRX17208371) in Project PRJNA873275 (accessed on 16 April 2025). Newly generated processed data, including the AS2/LOB protein full-length sequences of *A. officinalis*, detailed information of *AoAS* genes, the specific amino acid sequences of 15 motifs, *Cis*-acting element analysis of *AoAS* genes, duplication type of *AoAS* genes, list of PPI networks of *Ao*AS proteins and analysis of the expression pattern of *AoAS* genes in the stems of asparagus at different growth stages and under drought stress, are provided in the Supplementary Materials.

## Supplementary Materials

The following supporting information can be downloaded at https://www.mdpi.com/article/10.3390/genes16121411/s1: Table S1: The AS2/LOB protein full-length sequences of *A. officinalis* identified in this study. Table S2: Detailed information on *AoAS* genes. Table S3: The specific amino acid sequences of 15 motifs. Table S4: *Cis-*acting element analysis of *AoAS* genes. Table S5: Duplication type of *AoAS* genes. Table S6: List of PPI network of *Ao*AS proteins. Table S7: Analysis of the expression pattern of *AoAS* genes in the stems of asparagus at different growth stages and under drought stress.
